# Supplementary material for: Causal Relationship between Aging and Anorexia Nervosa: A White-Matter-Microstructure-Mediated Mendelian Randomization Analysis
Source: Biomedicines. 2024 Aug 16;12(8):1874. doi: 10.3390/biomedicines12081874 (PMC11351342; doi:10.3390/biomedicines12081874)
Supplement: Supplementary file 1 [file biomedicines-12-01874-s001.zip › Supplementary Figure/Figure S4/Singe SNP plot of MR analysis for TL to ubm-b-2121.pdf]

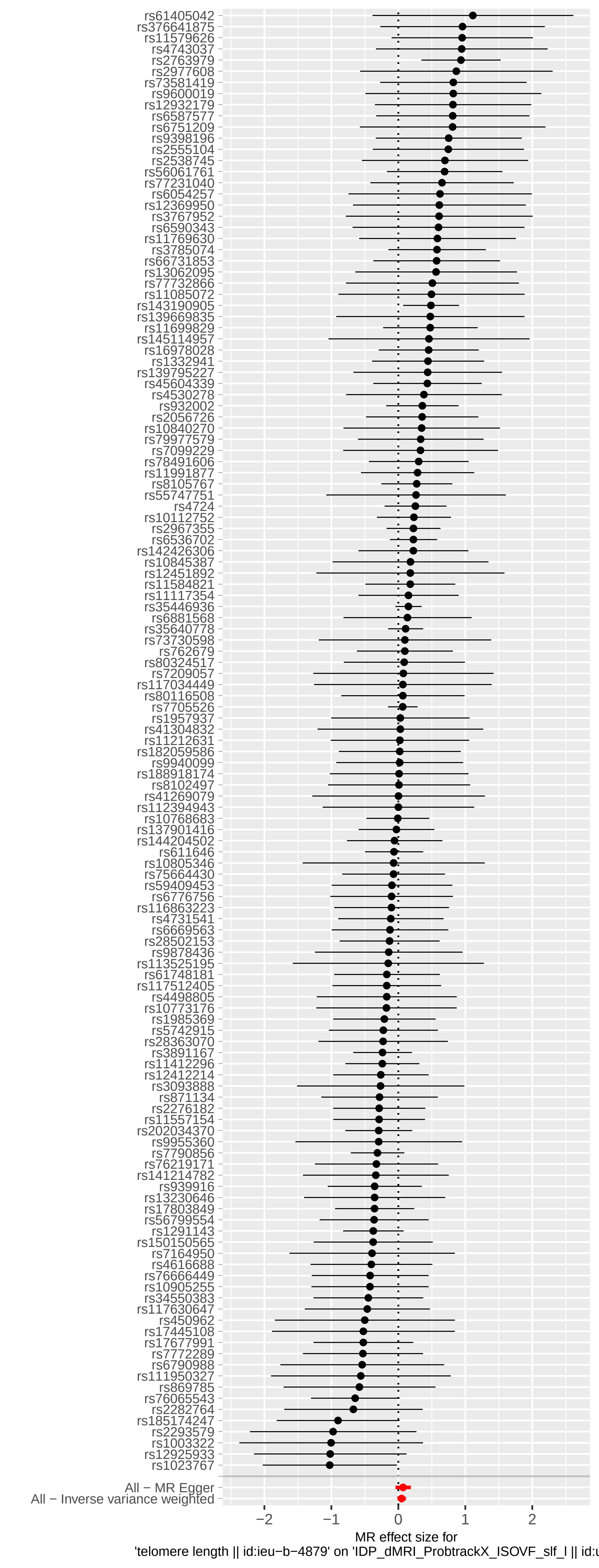
rs61405042
rs376641875
rs11579626
rs4743037
rs2763979
rs2977608
rs73581419
rs9600019
rs12932179
rs6587577
rs6751209
rs9398196
rs2555104
rs2538745
rs56061761
rs77231040
rs6054257
rs12369950
rs3767952
rs6590343
rs11769630
rs3785074
rs66731853
rs13062095
rs77732866
rs11085072
rs143190905
rs139669835
rs11699829
rs145114957
rs16978028
rs1332941
rs139795227
rs45604339
rs4530278
rs932002
rs2056726
rs10840270
rs79977579
rs7099229
rs78491606
rs11991877
rs8105767
rs55747751
rs4724
rs10112752
rs2967355
rs6536702
rs142426306
rs10845387
rs12451892
rs11584821
rs11117354
rs35446936
rs6881568
rs35640778
rs73730598
rs762679
rs80324517
rs7209057
rs117034449
rs80116508
rs7705526
rs1957937
rs41304832
rs11212631
rs182059586
rs9940099
rs188918174
rs8102497
rs41269079
rs112394943
rs10768683
rs137901416
rs144204502
rs611646
rs10805346
rs75664430
rs59409453
rs6776756
rs116863223
rs4731541
rs6669563
rs28502153
rs9878436
rs113525195
rs61748181
rs117512405
rs4498805
rs10773176
rs1985369
rs5742915
rs28363070
rs3891167
rs11412296
rs12412214
rs3093888
rs871134
rs2276182
rs11557154
rs202034370
rs9955360
rs7790856
rs76219171
rs141214782
rs939916
rs13230646
rs17803849
rs56799554
rs1291143
rs150150565
rs7164950
rs4616688
rs76666449
rs10905255
rs34550383
rs117630647
rs450962
rs17445108
rs17677991
rs7772289
rs6790988
rs111950327
rs869785
rs76065543
rs2282764
rs185174247
rs2293579
rs1003322
rs12925933
rs1023767
All − MR Egger
All − Inverse variance weighted
−2
−1
0
1
2
MR effect size for
'telomere length || id:ieu−b−4879' on 'IDP_dMRI_ProbtrackX_ISOVF_slf_l || id:u
